# Clinical Study of Autonomic Dysfunction in Patients With Anti-NMDA Receptor Encephalitis

**DOI:** 10.3389/fneur.2021.609750

**Published:** 2021-02-05

**Authors:** Lulu Yan, Shuang Zhang, Xiaoxue Huang, Yao Tang, Jun Wu

**Affiliations:** Department of Neurology, The First Affiliated Hospital of Zhengzhou University, Zhengzhou, China

**Keywords:** anti-NMDA receptor encephalitis, anti-NMDA antibody, autonomic dysfunction, severity of disease, prognosis

## Abstract

**Objectives:** Autonomic dysfunction is a common symptom of anti-N-methyl-D-aspartate receptor (NMDAR) encephalitis; however, it has been poorly researched. The purpose of this study was to compare the clinical features, tumor occurrence, intensive care unit (ICU) admission, mechanical ventilation, imaging assessment, cerebrospinal fluid examination, disease severity, and immunotherapy in patients with anti-NMDAR encephalitis with or without autonomic dysfunction.

**Methods:** A retrospective study of anti-NMDAR encephalitis patients diagnosed between January 2016 and April 2020 was performed at the First Affiliated Hospital of Zhengzhou University. Patients were divided into two groups according to whether they had autonomic dysfunction, and their clinical features, treatment, and prognosis were compared.

**Results:** A total of 119 patients with anti-NMDAR encephalitis were included in this study. Seventy-three patients (61.3%) had autonomic dysfunction, while the remaining 46 (38.7%) did not. Sinus tachycardia (69.9%) was the autonomic dysfunction with the highest incidence, while the incidences of symptoms including constipation, central hypopnea, and others gradually decreased. Compared to the group without autonomic dysfunction, the prevalence of the main clinical symptoms such as epileptic seizure (*P* = 0.003), involuntary movement (*P* = 0.028), and decreased consciousness (*P* < 0.001) were higher in the group with autonomic dysfunction, which also more frequently presented with complications such as pulmonary infection (*P* < 0.001) and abnormal liver function (*P* = 0.001). Moreover, the rates of ICU admission (*P* < 0.001) and mechanical ventilation (*P* = 0.001), as well as the modified Rankin scale (mRS) scores at admission (*P* < 0.001), maximum mRS scores during the course of disease (*P* < 0.001), and mRS scores at discharge (*P* < 0.001) were higher in the patients with autonomic dysfunction than in those without. The number of patients in the autonomic dysfunction group who underwent ≥2 immunotherapies was also higher than that in the group without autonomic dysfunction (*P* < 0.001).

**Conclusion:** Sinus tachycardia is the most common type of autonomic dysfunction in anti-NMDAR encephalitis. Compared to patients without autonomic dysfunction, those with autonomic dysfunction had a higher incidence of epilepsy, involuntary movements, decreased consciousness, pulmonary infections, abnormal liver function, ICU admissions, and mechanical ventilation; moreover, the severity of the disease was greater, and their prognosis worse. Therefore, such patients require intensive immunotherapy.

## Introduction

Autoimmune encephalitis (AE) refers to a non-infectious encephalitis that is mediated by autoimmune mechanisms. Anti-N-methyl-D-aspartate receptor (NMDAR) encephalitis is the most common AE (1). Its pathogenic mechanism is based on *in vivo* binding of anti-NMDAR antibodies to the extracellular NR1 subunit of the NMDAR, thereby disrupting the normal excitatory transmission in glutamatergic neurons and causing neuronal damage by inhibiting the localization and accumulation of NMDAR on neuronal cell membranes (2). In addition to non-specific prodromal symptoms such as fever, headache, nausea, and vomiting, the main clinical manifestations of encephalitis include abnormal mental behavior or cognitive impairment, seizures, dyskinesia or involuntary movement, dysphasia, decreased consciousness, and autonomic dysfunction (3). Autonomic dysfunction is a common clinical symptom of anti-NMDAR encephalitis; however, at present, there are few studies and with conflicting results, which explore its effects on patients' condition and prognosis (4–6). Therefore, this study aimed to explore the effects of autonomic dysfunction on clinical features, imaging and laboratory examination, disease severity, and treatment in patients with anti-NMDAR encephalitis to provide a future reference for the diagnosis and treatment of anti-NMDAR encephalitis.

## Methods

This retrospective study was approved by the Research Ethics Committee of the First Affiliated Hospital of Zhengzhou University (Zhengzhou, China) and carried out in compliance with the Declaration of Helsinki. Informed consent was obtained from each participant in the study.

### Study Population

Anti-NMDAR encephalitis patients who were diagnosed between January 1, 2016 and April 30, 2020 were retrospectively analyzed at the First Affiliated Hospital of Zhengzhou University. The inclusion criteria (1) were as follows: (1) after reasonable exclusion of other diseases, one or more symptoms belonged to the following six symptom groups: abnormal mental behavior or cognitive impairment; speech disorder (obsessive-compulsive speech, reduced speech, silence, and others); seizures; dyskinesia/involuntary movement; decreased level of consciousness; autonomic dysfunction, or central ventilation deficiency; and (2) Cerebrospinal fluid (CSF) that tested postivie for anti-NMDAR antibodies. On admission, the patients underwent a detailed consultation, neurological and physical examination, and laboratory and imaging examinations. The exclusion criteria were: (1) patients lacking key clinical data and (2) patients with heart, lung, kidney, or other multi organ dysfunctions.

### Data Collection

Demographic characteristics including age and sex, and clinical data including prodromal symptoms, first symptoms, main clinical manifestations, complications during hospitalization, concomitant tumors, intensive care unit (ICU) admission, mechanical ventilation, baseline results of head magnetic resonance imaging (MRI), detection of anti-NMDAR antibodies in blood and CSF, routine biochemical and cytological CSF tests for the first time on admission, and immunotherapy were assessed and recorded. The modified Rankin scale (mRS) score was used to evaluate neurological function (7). The mRS scores at admission, maximum course of disease, and at discharge were evaluated and recorded. The Glasgow Coma Score (GCS) was used to assess the state of consciousness (8). The normal reference range of each index set by our laboratory was as follows: in CSF cytological examination, the normal value of white blood cell count was 0–5 × 10^6^ /L, oligoclonal bands were negative, and the normal range of albumin quotient 0–9. Based on search results obtained from the electronic medical record system, the patients were divided into two groups according to whether symptoms of autonomic dysfunction occurred during the course of the disease: one with autonomic dysfunction and another without.

In this study, the definition of the specific symptoms of autonomic dysfunction was based on previously published literature reports (9–12), including sinus tachycardia (cardiac disorders, fever, pain, and anemia were excluded, and a heart rate >100 beats/min on electrocardiogram monitoring), bradycardia, hypersalivation, central hypopnea, hypotension, hyperhidrosis, constipation, uroclepsia, uroschesis, and fervescence, among others.

The mental and behavioral abnormalities considered in this study include irritability, balderdash, personality changes, emotional disorders, volition disorders, behavior disorders, perception disorders, and thought disorders, among others. Cognitive impairment includes lags in response, memory, comprehension, calculation, orientation, and weak judgment and executive functioning, among others. Decreased consciousness was determined by observing the patient's state of consciousness during hospitalization and calculating the GCS score. A GCS score ≤14 signified a decrease in consciousness. The assessment of central hypopnea was based on the presence of shortness of breath, decreased oxygen saturation, and increased partial pressure of carbon dioxide, as well as the need for a nasal catheter, oxygen mask, endotracheal intubation, or tracheotomy after the elimination of respiratory diseases during the course of the disease. Immunotherapy included hormones, plasma exchange/plasma immunoadsorption, gamma globulin, azathioprine, cyclophosphamide, mycophenolate mofetil, and rituximab.

### Statistical Analysis

Statistical analyses were performed using the software SPSS 25.0 (IBM Corporation, Armonk, NY, USA). Data with normal distribution were expressed as the means ± standard deviations, whereas data with non-normal distribution were expressed as medians (interquartile ranges, IQR). The Student's *t*-test was used for intergroup comparisons of data with a normal distribution, whereas the Mann–Whitney *U*-test was employed for those with a non-normal distribution. Countable data were expressed as frequencies (percentages) [*n* (%)] and compared using the Chi-square test, continuous correction Chi-square test, or Fisher's exact probability method. The significance level was set at *p* < 0.05.

## Results

### Incidence of Different Symptoms of Autonomic Dysfunction

A total of 119 patients with a definitive diagnosis of anti-NMDAR encephalitis were included in this study. Seventy-three patients (61.3%) had autonomic dysfunction, including sinus tachycardia (51 patients, 69.9%), constipation (41 patients, 56.2%), central hypopnea (26 patients, 35.6%), pollakiuria/uroclepsia (22, 30.1%), hypersalivation (eight patients, 11.0%), hyperhidrosis (eight patients, 11.0%), sinus bradycardia (six patients, 8.2%), hypotension (three patients, 4.1%), uroschesis (three patients, 4.1%), and fervescence (two patients, 2.7%) (Table 1).

**Table 1:** Clinical manifestation and incidence of autonomic dysfunction.

**Autonomic dysfunction**	**Patients (%)**
Sinus tachycardia	51 (69.9)
Constipation	41 (56.2)
Central hypopnea	26 (35.6)
Pollakiuria / uroclepsia	22 (30.1)
Hypersalivation	8 (11.0)
Hyperhidrosis	8 (11.0)
Sinus bradycardia	6 (8.2)
Hypotension	3 (4.1)
Uroschesis	3 (4.1)
Fervescence	2 (2.7)

### Demographic and General Clinical Characteristics

The demographic and general clinical characteristics of the patients are summarized in Table 2. The study included 119 patients, including 67 men and 52 women, with a median age of 28 years (range: 1–74 years). There was no significant difference in the sex ratio and age composition between the two groups.

**Table 2:** Comparison between two groups of patients with or without autonomic dysfunction.

	**Group with autonomic dysfunction (*n* = 73)**	**Group without autonomic dysfunction (*n* = 46)**	***X*^2^/*Z***	***P***
Male:female	39:34	28:18	0.636	0.425
Age, mean ±*SD*, year	29.41 ± 14.718	29.65 ± 14.649	−0.087	0.931
Prodromal symptoms, *n* (%)	42 (57.5)	30 (65.2)	0.697	0.404
**First symptoms**, ***n*** **(%)**				
Mental and behavioral abnormalities / cognitive impairment	44 (60.3)	28 (60.9)	0.004	0.948
Epilepsy	17 (23.3)	13 (28.3)	0.370	0.543
**Main clinical manifestations**, ***n*** **(%)**				
Mental and behavioral abnormalities / cognitive impairment	64 (87.7)	41 (89.1)	0.058	0.810
Epilepsy	52 (71.2)	20 (43.5)	9.096	0.003
Involuntary movement	32 (43.8)	11 (23.9)	4.853	0.028
Decreased consciousness	68 (93.2)	20 (43.5)	36.142	<0.001
Speech disturbances	28 (38.4)	10 (21.7)	3.585	0.058
**Complications**, ***n*** **(%)**				
Pulmonary infection	55 (75.3)	15 (32.6)	21.275	<0.001
Abnormal liver function	52 (71.2)	19 (41.3)	10.503	0.001
Venous thrombosis	16 (21.9)	4 (8.7)	3.528	0.060
Bacteremia	6 (8.2)	0 (0.0)	2.450	0.118
Tumor, *n* (%)	6 (8.2)	4 (8.7)	0.000	1.000
ICU admission, *n* (%)	59 (80.8)	5 (10.9)	55.551	<0.001
Mechanical ventilation, *n* (%)	16 (21.9)	0 (0.0)	11.648	0.001
**Location of MRI abnormal signal**				
Cerebral cortex or limbic system: other brain regions	12:13	14:13	0.077	0.781
**Cerebrospinal fluid examination**, ***n*** **(N, %)**				
Increased WBC count	49 (63, 77.8)	28 (43, 65.1)	2.026	0.151
Oligoclonal bands	13 (55, 23.6)	8 (36, 22.2)	0.025	0.876
Albumin quotient	14 (55, 25.5)	15 (37, 40.5)	2.332	0.127
Serum anti-NMDAR antibody, *n* (N, %)	31 (53, 58.5)	11 (20, 55.0)	0.072	0.788
CSF anti-NMDAR antibody, *n* (N, %)	73 (73, 100.0)	46 (46, 100.0)	-	-
**mRS score, median (IQR)**				
Admission mRS score	3 (3, 4)	2 (2, 3)	−4.423	<0.001
Maximum mRS score in the course of disease	5 (4, 5)	3 (2.75, 3.25)	−7.725	<0.001
Discharge mRS score	3 (2, 4)	2 (1, 2)	−6.704	<0.001
**Immunotherapy**, ***n*** **(%)**				
No or one kind of immunotherapy	17 (23.3)	27 (58.7)	15.182	<0.001
Two or more immunotherapies	56 (76.7)	19 (41.3)		

*ICU, Intensive Care Unit; MRI, magnetic resonance imaging; NMDAR, N-methyl-d-aspartate receptor; CSF, cerebrospinal fluid; WBC, white blood cell count; mRS, modified Rankin scale*.

In this study, a total of 52 patients in the group with autonomic dysfunction developed epileptic seizures, including 33 with generalized tonic-clonic seizures, 12 with simple partial motor seizures, five with complex partial seizures, and two with partial motor seizures followed by generalized seizures. A total of 20 patients in the group without autonomic dysfunction developed epileptic seizures, including 12 who developed generalized tonic-clonic seizures, four with complex partial seizures, one with complex partial seizures followed by generalized seizures, two with partial motor seizures, and one with partial sensory seizures. Involuntary movements included tremors in the face, limbs, or torso. In addition, there were six cases with concomitant tumors [four with ovarian teratoma [female], one with gallbladder carcinoma (male), and one with meningioma (female)] in the group with autonomic dysfunction. In the group without autonomic dysfunction, there were four cases with concomitant tumors [two with ovarian teratoma (female), one with anterior mediastinal teratoma (female), and one with pharyngeal non-Hodgkin's lymphoma (male)].

In total, 73 and 46 patients were included in the group with autonomic dysfunction and the group without, respectively. Fifty-two and 20 patients had epilepsy as one of among the main clinical manifestations in the group with autonomic dysfunction and the group without, respectively (*P* = 0.003). Thirty-two patients in the group with autonomic dysfunction had involuntary movement as one of the main clinical manifestations, compared with 11 in the group without autonomic dysfunction (*P* = 0.028). Sixty-eight patients in the group with autonomic dysfunction had a decreased consciousness as one of the main clinical manifestations, compared with 20 patients in the group without autonomic dysfunction (*P* < 0.001). In terms of complications, pulmonary infection occurred in 55 patients in the group with autonomic dysfunction and 15 patients in the group without (*P* < 0.001). In addition, 52 patients in the group with autonomic dysfunction had abnormal liver function, while 19 patients did in the group without autonomic dysfunction (*P* = 0.001). Moreover, 59 patients in the group with autonomic dysfunction had been admitted to the ICU, while only five in the group without autonomic dysfunction (*P* < 0.001) were admitted. Sixteen patients in the group with autonomic dysfunction required mechanical ventilation, while none required it in the group without autonomic dysfunction (*P* = 0.001).

The frequency of other clinical features, such as prodromal symptoms, first symptoms, main clinical manifestations (mental and behavioral abnormalities, cognitive impairment, and speech disturbances), complications during hospitalization (venous thrombosis and bacteremia), tumor presentation rate, rate of anti-NMDAR antibody positivity in serum and CSF, and abnormal CSF findings were not significantly different between the two groups. In this study, MRI results revealed abnormal signals in single or multiple parts of the brain, such as the cerebellum or cerebral cortex, frontal lobe, temporal lobe, parietal lobe, and limbic system. We divided the abnormal MRI findings into the limbic system and/or cerebral cortex and other parts, and compared them. No significant difference was found in the abnormal MRI findings between patients with or without autonomic dysfunction.

We also compared the severity of disease and treatment strategies between the two groups. Compared to the group without autonomic dysfunction, the disease severity was greater in patients with autonomic dysfunction, and the admission mRS score (median, 3 vs. 2, *P* < 0.001), maximum mRS scores during the course of disease (median, 5 vs. 3, *P* < 0.001), and discharge mRS score (median, 3 vs. 2, *P* < 0.001) were higher. In addition, patients in the group with autonomic dysfunction were more likely to receive two or more immunotherapies compared with those in the group without autonomic dysfunction (*P* < 0.001).

## Discussion

Autonomic dysfunction, which includes sinus tachycardia, bradycardia, hypersalivation, central hypopnea, hypotension, hyperhidrosis, constipation, pollakiuria/uroclepsia, uroschesis, and fervescence (9–12) is a common clinical manifestation of anti-NMDAR encephalitis. Autonomic dysfunction of anti-NMDAR encephalitis may be associated with damage of autonomic nerve centers (13). A previous study has reported that autonomic dysfunction is the first symptom in patients with anti-NMDAR encephalitis (14). Autonomic dysfunction can occur at different stages of the disease, sometimes even throughout the disease, and may even affect prognosis. However, the autonomic dysfunction of anti-NMDAR encephalitis has received little attention. Studying the autonomic dysfunction of anti-NMDAR encephalitis may help us to better understand anti-NMDAR encephalitis, evaluate the therapeutic effects, and assess the prognosis, which was the purpose of this study.

In terms of incidence and demographic characteristics, a small study (10) found that the incidence of autonomic dysfunction in anti-NMDAR encephalitis is 69%. In the present study, 73 patients (61.3%) had autonomic dysfunction, similar to the results of this previous study. Our results also showed that the most common type of autonomic dysfunction was sinus tachycardia, followed by constipation, central hypopnea, pollakiuria/uroclepsia, hypersalivation, hyperhidrosis, sinus bradycardia, hypotension, uroschesis, and fervescence. After active immunotherapy, tumor resection, and other symptomatic treatments, the symptoms of autonomic dysfunction in the vast majority of patients can disappear.

In terms of MRI results, we found no significant difference in abnormal rates between the two groups. However, it is worth mentioning that anti-NMDAR encephalitis mainly involves neurons and synaptic structures, resulting in ion channel damage, which further leads to dysfunction of the cerebral cortex and neural networks, but the current 3.0T MRI plain scan is often unable to comprehensively and accurately reflect these changes. In the future, the use of high field intensity and/or functional MRI may contribute to the in-depth understanding and accurate assessment of the severity of NMDAR encephalitis.

After a systematic review and analysis of the data, we found significant differences between the two groups in terms of clinical manifestations and complications, as well as disease severity and immunotherapy.

The incidence of epilepsy, involuntary movement, and decreased consciousness in the group with autonomic dysfunction was significantly higher than in the group without autonomic dysfunction. Anti-NMDAR encephalitis lesions often involve the limbic system, which not only involve the autonomic nerve centers of respiratory function, heartbeat, gastrointestinal function, and other important visceral activities, but are also a common region of origin of seizures (15, 16). When anti-NMDAR encephalitis lesions involve the corpus striatum, amygdaloid body, and other extrapyramidal structures (17), it will lead to involuntary movements such as tremors, dance-like movements, and myoclonic symptoms (17, 18), which are closely associated with autonomic nerve function. The cerebral cortex and brain stem are involved in the regulation of autonomic nervous function and the level of consciousness (19, 20). There are complex connections between the epileptic, autonomic movement, and consciousness regulating brain regions and autonomic nervous function regulating centers. This may result in a higher incidence of epilepsy, involuntary movement, and decreased consciousness in patients with autonomic dysfunction.

Patients with anti-NMDAR encephalitis often have a variety of complications. In our study, we found that the incidence of pulmonary infection and abnormal liver function in patients with autonomic dysfunction was significantly higher than in patients without autonomic dysfunction. This may be due to the following: First, the autonomic neural networks are widely distributed in the brainstem, which also includes respiratory centers. When lesions spread to the brainstem, autonomic nerve function and respiratory function disorders can simultaneously occur (21). Second, autonomic nervous function is also closely associated with liver function. Studies have found that damage to the ventromedial hypothalamus can lead to changes in liver cells, and autonomic nervous function can be abnormal when the liver is damaged (22, 23).

Compared with the group without autonomic dysfunction, patients with autonomic dysfunction more frequently required ICU admissions and mechanical ventilation. Moreover, they also had higher mRS scores, maximum mRS scores during the course of the disease, and mRS scores at discharge, which means that their condition was more serious at admission, more prone to deterioration during the course of treatment and had a relatively poor short-term prognosis. The reason may be that patients with autonomic dysfunction are more likely to suffer blood pressure regulation disorders, arrhythmias, and sudden death. Respiratory and urinary dysfunction also increase the risk of lung and urinary tract infections, respectively. Various conditions can lead to serious illness and poor prognosis. This study also showed that the proportion of patients with autonomic dysfunction undergoing two or more kinds of immunotherapy was significantly higher than that of those without autonomic dysfunction, which also indirectly indicates that patients with autonomic dysfunction have a relatively more serious condition and are more difficult to treat.

In summary, our study showed that patients with anti-NMDAR encephalitis with autonomic dysfunction have a higher incidence of epilepsy, involuntary movements, and decreased consciousness over the course of the disease, and are more prone to have a pulmonary infection and abnormal liver function. Moreover, their initial condition is more serious, the tendency of deterioration more obvious, and the prognosis relatively poor. Therefore, in the process of diagnosis and treatment, attention should be paid to the autonomic dysfunction of patients with anti-NMDAR encephalitis. The condition of such patients should be closely monitored, and timely symptomatic treatment such as anti-epilepsy, anti-infection, liver protection, and strengthened immunotherapy should be provided. However, there are some limitations to this study: First, when defining the presence of autonomic dysfunction, this study refers to some references to define whether there is autonomic dysfunction, which involves a certain degree of subjectivity (9–12). Strictly speaking, more objective methods have been used for the assessment of autonomic nervous system function, such as heart rate variability, electrodermal activity, dynamic pupillometry, and cardiovascular autonomic function testing (5, 24–26). These methods are more objective and will likely be applied to improve our research in the future. Second, although this study conducted a rigorous statistical analysis of patients with anti-NMDAR encephalitis in our center from January 2016 to April 2020, the results may be limited due to the single-center, small-sample retrospective nature of the study.

In conclusion, this study constitutes a preliminary clinical exploration of anti-NMDAR encephalitis, which not only provides inspiration and reference for follow-up research, but also lays a foundation for multicenter, large-sample clinical research in the future. We aim to conduct more well-designed studies in the future to provide new methods and protocols for the diagnosis and treatment of NMDAR encephalitis.

## Data Availability Statement

The raw data supporting the conclusions of this article will be made available by the authors, without undue reservation.

## Ethics Statement

The studies involving human participants were reviewed and approved by the Research Ethics Committee of the First Affiliated Hospital of Zhengzhou University (Zhengzhou, China). Written informed consent to participate in this study was provided by the participants' legal guardian/next of kin.

## Author Contributions

L-LY wrote the main manuscript, analyzed the data, and prepared Tables 1, 2. L-LY, SZ, X-XH, YT, and JW collected the data. JW designed the research, lead the research group, and arranged the work of all authors. All authors have reviewed the manuscript.

## Conflict of Interest

The authors declare that the research was conducted in the absence of any commercial or financial relationships that could be construed as a potential conflict of interest.
